# *Drosophila* GAGA factor polyglutamine domains exhibit prion-like behavior

**DOI:** 10.1186/1471-2164-14-374

**Published:** 2013-06-03

**Authors:** Muhammad Tariq, Renee Wegrzyn, Saima Anwar, Bernd Bukau, Renato Paro

**Affiliations:** 1 Department of Biology, SBA School of Science and Engineering, Lahore University of Management Sciences, Lahore 54792, Pakistan; 2 Department of Biosystems Science and Engineering, ETH Zurich, Mattenstrasse 26, Basel 4058, Switzerland; 3 Faculty of Science, University of Basel, Basel 4056, Switzerland; 4 Zentrum für Molekulare Biologie Heidelberg, Im Neuenheimer Feld 282, Heidelberg 69120, Germany

**Keywords:** GAGA factor, Epigenetics, Prion-like, Chromatin

## Abstract

**Background:**

The Drosophila GAGA factor (GAF) participates in nucleosome remodeling to activate genes, acts as an antirepressor and is associated with heterochromatin, contributing to gene repression. GAF functions are intimately associated to chromatin-based epigenetic control, linking basic transcriptional regulation to heritable long-term maintenance of gene expression. These diverse functions require GAF to interact with different partners in different multiprotein complexes. The two isoforms of GAF depict highly conserved glutamine-rich C-terminal domains (Q domain), which have been implicated in complex formation.

**Results:**

Here we show that the Q domains exhibit prion-like properties. In an established yeast test system the two GAF Q domains convey prion activities comparable to well known yeast prions. The Q domains stably maintain two distinct conformational states imposing functional constraints on the fused yeast reporter protein. The prion-like phenotype can be reversibly cured in the presence of guanidine HCl or by over-expression of the Hsp104 chaperone protein. Additionally, when fused to GFP, the Q domains form aggregates in yeast cells.

**Conclusion:**

We conclude that prion-like behavior of the GAF Q domain suggests that this C-terminal structure may perform stable conformational switches. Such a self-perpetuating change in the conformation could assist GAF executing its diverse epigenetic functions of gene control in *Drosophila*.

## Background

The GAGA factor (GAF) of *Drosophila* is a ubiquitous transcription factor that plays important roles in multiple processes ranging from regulation of gene expression to the structural organization of heterochromatin and chromatin remodeling [1-5]. Genetically, GAF is classified as a member of the Trithorax group proteins (TrxG) counteracting the silencing of Polycomb group proteins (PcG) by maintaining an epigenetically heritable active state of gene expression [6]. However, the identified biochemical interactions with a variety of chromatin remodeling complexes and mutant analyses indicate a much broader role for GAF [7-9]. How such divergent functions of GAF might be acquired and controlled still remains elusive.

**Figure 1:**
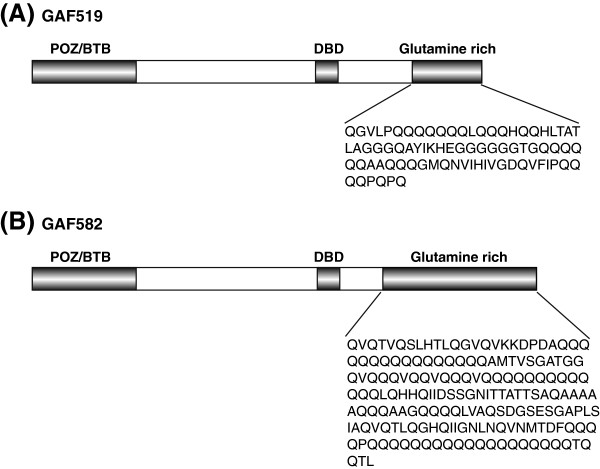
**Schematic diagram of GAGA factor (GAF) isoforms in Drosophila containing 519 amino acids (GAF519) and 582 amino acids (GAF582).** The two isoforms, GAF519 (**A**) and GAF582 (**B**), only differ in the glutamine rich stretch within the C terminal region of proteins. The specific amino acid sequence of glutamine rich (Q) domains are shown below each isoform. In addition to Q domains, POZ/BTB domain and DBD (DNA binding domains) in both isoforms are also highlighted.

In *Drosophila*, multiple GAF isoforms are encoded from a single gene termed *Trithorax-like (Trl)*[6,10,11]. All *Trl* splice forms characterized so far, contain two open reading frames for a protein of 519 (GAF519) or 582 amino acid (GAF582) residues, respectively [10-12]. Both GAF isoforms contain 3 recognizable and evolutionarily highly conserved domains, a POZ/BTB domain, a zinc finger DNA binding domain and a glutamine-rich region referred to here as the GAF-Q or polyglutamine (polyQ) domain [13,14]. The isoforms are highly identical in their N-terminal part but they differ in the length of the C-terminal glutamine-rich regions (Figure 1) [10,11]. The POZ/BTB domain has been shown to function as a protein-protein interaction domain and the DBD (DNA binding domain) domain is important for sequence recognition and DNA binding activity [14]. Although Q domains found in different transcription factors were suggested to be associated with transcriptional control [13,15,16], in one study GAF-Q was shown to be dispensable for chromatin binding and transcriptional activation [12,17]. However, other studies assign the transcriptional activity to the C-terminal polyQ domain [18]. Interestingly, *in vitro* studies have suggested that the GAF-Q domain of GAF519 facilitates multimerization, which may explain the multimeric distribution of GAF observed *in vivo*[19]. Additionally, the GAF519 polyQ domain was shown to be essential for the formation of long unbranched amyloid fibers *in vitro*[17]. The amyloid fibers formed by the GAF519 resemble fibers formed by Sup35, the prion determinant of the yeast prion [*PSI*^*+*^] [20].

**Figure 2:**
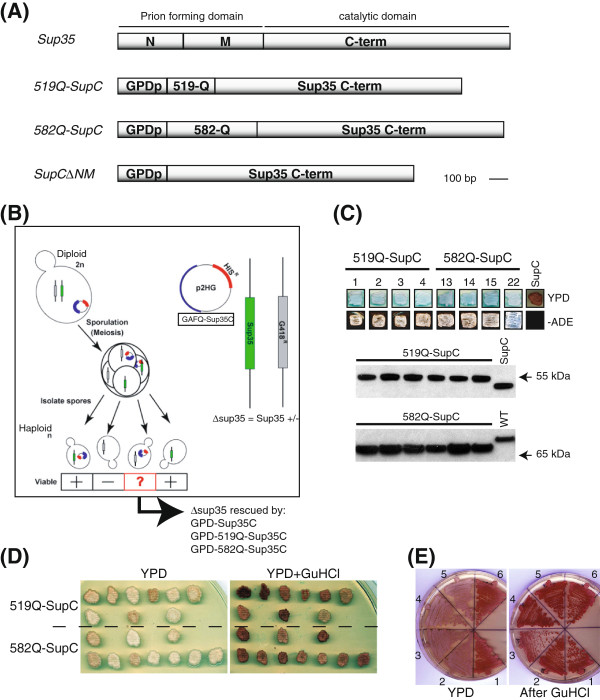
***Drosophila *GAF polyglutamine domains (519Q and 582Q) substitute for prion domain of Sup35p in yeast.** (**A**) Full length Sup35 regions encoding N, M and C term modules are indicated where  N and M are known prion domain. *GAF519* and *GAF582* sequences encoding Q domains (519Q and 582Q) were fused to sequences encoding the C-terminal domain of Sup35p. The C-terminal of Sup35p alone (SupC) was used as a control. (**B**) Schematic picture depicting *GAFQ-SupC* chimeric (*519Q-SupC and 582Q-SupC*) constructs with *HIS* selectable marker tranfected in a diploid [*PSI*^*+*^] yeast strain, which was deleted for one copy of endogenous *SUP35* indicated by *G418* selectable marker. After induction of sporulation in transformants, the resulting haploids were selected on -His and G418 and analyzed for viability of individual spores. (**C**) The *sup35*∆ haploids with 519Q-SupC and 582Q-SupC fusions grow on YPD and -Ade media whereas SupC∆NM (SupC) containing haploid does not grow on -Ade and appear as red. The growth on -Ade plates indicates a nonsense suppression prion-like phenotype similar to [*PSI*^+^]. Expression levels of GAFQ-SupC fusions (519Q-SupC 57 kDa, 582Q-SupC 67 kDa, SupC∆NM 48 kDa) in individual haploids analyzed by Western blot with anti-Sup35 antibody recognizing only the C-terminal of Sup35. Total extract from wild type (WT) yeast shows endogenous Sup35 protein. (**D**) The prion-like phenotype of *GAFQ-SupC* expressing individual haploid cells is shown to be cured by 5 mM guanidine HCl (GuHCl), indicated by reversion of pink cells to red. (**E**) Streaking of GuHCl treated haploids show stability of non-sense suppression in two independent haploids of 519Q-SupC (2, 3) and 582Q-SupC (4, 5) taken from (**D**) which appear pink on YPD as compared to red colored haploids containing SupC∆NM (1,6). Both 519Q-SupC (2, 3) and 582Q-SupC (4, 5) haploids show curing after GuHCl and appear red as similar to SupC∆NM (1,6) haploids.

Like most yeast prions, Sup35 has a well characterized glutamine or asparagine (Q/N) rich region, which is known as the prion domain (Figure 2A) [20,21]. It enables Sup35 to exist in distinct physical and functional states that are interconvertible and heritable, i.e. a [*psi*^*-*^] soluble, functional state and a [*PSI*^*+*^] aggregated, non-functional state [20]. In [*psi*^*-*^] cells, the Sup35 protein acts as a translation termination factor that, together with Sup45, recognizes stop codons and terminates translation. In contrast, in [*PSI*^*+*^] cells the Sup35 is unable to efficiently participate in translation termination as most of Sup35 protein is sequestered in self-replicating prion multimers [20]. Interestingly, the existence of chromatin associated proteins Swi1 and Cyc8 in distinct physical and functional states, reminiscent of prion-like behavior, was discovered in yeast [22,23]. The glutamine-rich regions of Swi1 and Cyc8 were shown to be essential for prion-like behavior of these proteins and modulation of global gene expression patterns which are epigenetically inherited [22,23]. Although a large number of proteins in eukaryotic proteins have long Q-rich tracts, similar to those found in the prion domains of yeast prions [24-26], only a few are characterized to have properties similar to prions [27,28].

Expansion of polyQ domains is known to contribute to heritable alterations of protein conformation which is associated with prion proteins [20,21]. Hence, we tested whether the Q domains of the GAF isoforms, GAF519 and GAF582, could act as prion-like domains using established validation tools in the yeast *Saccharomyces cerevisiae*. We replaced the N-term prion-forming domain of Sup35 with the GAF519 and GAF582 Q domains (GAF-Q) and fused it to the C-terminal portion of the Sup35 in order to test whether the chimeric proteins were able to induce a prion-like state as demonstrated for a variety of other potential prion forming domains [29-32]. The resulting GAFQ-SupC fusion proteins were able to rescue the lethality of a Sup35 deletion strain. Expression of GAFQ-SupC led to the appearance of a stably maintained nonsense suppression phenotype in a small proportion of cells, in a manner similar to the appearance of [*PSI*^*+*^] in cells over-expressing Sup35. This prion-like phenotype of the GAFQ-SupC expressing cells could be reversibly cured in the same manner as the [*PSI*^*+*^] prion by growing the cells in the presence of guanidine HCl (GuHCl) or by over-expression of the Hsp104 chaperone protein. Furthermore, when fused to GFP, the GAF519 and GAF582 polyQ domains formed aggregates in yeast cells. In corroboration, sedimentation analysis using differential centrifugation revealed that GAFQ-SupC fusions are aggregated in the same manner as the [*PSI*^*+*^] prion. These aggregates were cured by growing the cells in the presence of GuHCl. Finally, meiotic segregation analysis through tetrad dissection also revealed that the prion-like phenotype of GAF582SupC segregated in a non-Mendelian fashion. Importantly, prion-like behavior exhibited by meiotic progeny was also cured when grown in the presence of GuHCl. Such prion-like behavior of GAF polyQ domains in yeast suggest that polyQ domains in GAF may render a conformational switch which may help GAF perform its versatile functions in maintaining different epigenetic states of gene expression.

## Results and discussion

The GAF isoforms GAF519 and GAF582 share the same N terminal domain but have distinct glutamine (Q)-rich C termini of different lengths. The C terminal 80 amino acids of the GAF519 contains 39% Q residues whereas the C terminal 178 amino acids of the GAF582 comprises of 41% of Q residues in multiple repeats (Figure 1). The well characterized genetic assays based on the [*PSI*^*+*^] reporter system [33] for evaluation of prions in yeast were employed to examine if GAF519 and GAF582 polyQ domains (GAFQ) can act as prion-like domains. A colony color assay based on [*psi*^*-*^] and [*PSI*^*+*^] states of Sup35 can be employed to reproducibly monitor the prion-like behavior of Q-rich regions [20]. The non-prion, functional part of Sup35p i.e. SupC needed for translation termination has been used with fusions of Q-rich yeast protein regions to detect prion-like behavior [29,31,34]. In the case of the [*psi*^*-*^] state, cells that carry a premature stop codon in their *ADE1* gene (mutant designated *ade1-14*) do not make functional Ade1 and accumulate a metabolite making the colonies appear red on complete medium. In contrast, the [*PSI*^*+*^] cells are characterized by a read-through of the premature stop codon (nonsense suppression) in *ade1-14.* Functional Ade1 is produced as most of Sup35 protein is sequestered in self-replicating prion aggregates and is unable to participate in translation termination. [*PSI*^*+*^] cells therefore produce white colonies and can grow on adenine-deficient medium [33].

We generated DNA constructs replacing the prion domain of Sup35 with either GAF519 or GAF582 Q domains (GAF-Q) to monitor if GAF-Q can substitute for the Sup35 prion domain (Figure 2A). The non-prion, functional part of Sup35p (Sup35C) was fused with GAF519 and GAF582 Q-rich regions (GAF-Q) and the resultant fusion constructs were named as *519Q-SupC* and *582Q-SupC* (*GAFQ-SupC)* (Figure 2A). The *GAFQ-SupC* fusions and *SupC* alone (Figure 2A) under a constitutive promoter were transfected in a diploid [*PSI*^*+*^] yeast strain GT81 [35] in which we introduced a deletion of one copy of endogenous *SUP35* (Figure 2B). In strain GT81, the [*PSI*^*+*^] suppressible marker is *ade1-14* with a UGA premature stop codon. [*PSI*^*+*^] cells produce white colonies on complete media (YPD) and grow without adenine supplementation. In contrast [*psi*^*-*^] cells do not form colonies on adenine-deficient medium (−Ade) and are red on YPD [35]. The *GAFQ-SupC* containing diploid cells heterozygous for *Sup35* were induced for sporulation (Figure 2B) and resulting *sup35*∆ haploids were specifically monitored for viability by selecting on media supplemented with G418 and lacking histidine (−His), indicators for the *Sup35* deletion and plasmids carrying *GAFQ-SupC* fusions, respectively (Figure 2B). Further, the *sup35*∆ haploids were confirmed by detecting expression of endogenous Sup35 using antibody raised against the N-terminal (region containing prion domain) of Sup35 (Additional file 1: Figure S1). The haploids confirmed for deletion of Sup35 were also probed for expression of fusion proteins with an antibody raised against the C terminus of Sup35 (Figure 2C), which detected signals at 57 kDa and 67 kDa, the expected sizes of the 519Q-SupC and 582Q-SupC fusions, respectively (Figure 2C). The growth of *sup35*∆ haploids on YPD indicated that lethality based on the absence of Sup35 was rescued by both 519Q-SupC and 582Q-SupC fusions (Figure 2C). Importantly, the haploids expressing 519Q-SupC and 582Q-SupC fusions produced white colonies on YPD and were viable on -Ade medium (Figure 2C). This is indicative of a nonsense suppression prion-like phenotype caused by the read-through of a nonsense codon in *ade1-14* marker (Figure 2C), similar to [*PSI*^*+*^] cells. In contrast, *sup35*∆ haploids expressing *SupC∆NM* (SupC alone; Figure 2A) produced red colored colonies and did not grow on medium lacking adenine (Figure 2C). This clearly illustrates that polyQ domains of GAF519 and GAF582 may substitute the prion domain of Sup35 and exhibit behavior similar to the Sup35 prion domain.

Because all yeast prions characterized so far exhibit the ability to exist in two functionally distinct states that are heritable and interconvertible at low frequency [20] we next monitored the metastable behavior of GAFQ-SupC containing *sup35*∆ haploids. The existence of GAFQ-SupC in two different functional states could be visualized by the colony color assay by repeatedly streaking individual haploids on YPD plates and monitoring the appearance of red and white colored colonies. GAFQ-SupC expressing fusions in *sup35*∆ haploids led to the appearance of colonies which were metastable, resulting in the appearance of some red colonies after several generations, similar to the metastability of some [*PSI*^*+*^] prion phenotypes [20,33,36]. Reversible curing of prion phenotype is an important genetic criterion for analysis of prion proteins in yeast. The known yeast prions [*URE3*], [*PSI*^*+*^], [*PIN*^*+*^], [*SWI*^*+*^] and [*OCT*^*+*^] are cured by growth in the presence of guanidine HCl (GuHCl), which is suggested to inhibit heat shock protein 104 (Hsp104) [37,38]. The reversible curing of the prion-like phenotype of GAFQ-SupC expressing cells was demonstrated by the conversion of colonies from a white color on YPD to a red color on YPD plates supplemented with 5 mM guanidine HCl (GuHCl) (Figure 2D, E). Similar to the reversible curing of the [*PSI*^*+*^] phenotype [20], the GAFQ-SupC nonsense suppression phenotype (visible as white colonies) was cured by growth in the presence of GuHCl, resulting in the appearance of red colored colonies mimicking the [*psi*^*-*^] phenotype (Figure 2D), which remained stably cured when grown in the absence of GuHCl (Figure 2E). The colony color assay as well as reversible curing by GuHCl clearly illustrates that the GAF-Q domains have the ability to confer upon Sup35C a capacity to exist in distinct physical and functional states that are interconvertible and heritable.

**Figure 3:**
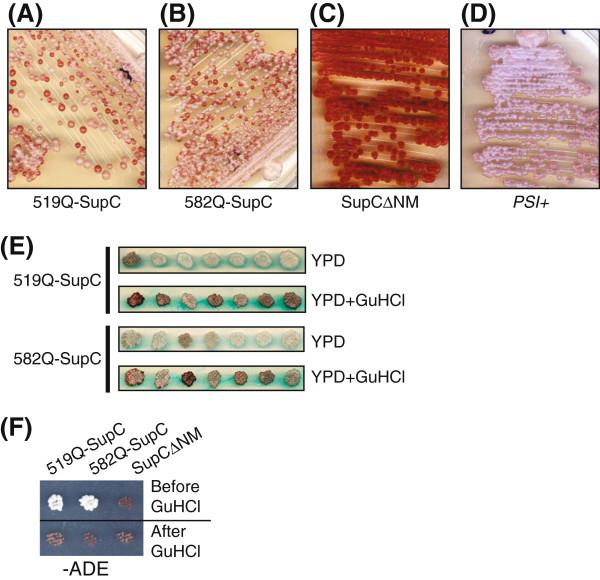
**GAFQ-SupC (519Q-SupC and 582Q-SupC) chimeric proteins exhibit prion-like behavior similar to [*PSI***^***+***^**] phenotype of Sup35p.** (**A**-**D**) Analysis of metastable behavior of GAFQ-SupC haploids is shown. Individual haploids containing 519Q-SupC (**A**) and 582Q-SupC (**B**) are shown with metastable behavior indicated by the presence of red and pink colored colonies as compared to controls; SupC-expressing cells (**C**) where only red colonies appeared and OT55 strain which show stable [*PSI*^*+*^] (**D**). The presence of pink colored colonies, due to nonsense suppression of the *ade1-14* reporter, was stably maintained upon restreaking, similar to the appearance of [*PSI*^*+*^]. (**E**) Individual pink/white colonies picked from (**A**) and (**B**) were grown on YPD and replica plated on YPD plus 5 mM GuHCl which resulted in curing of nonsense suppression phenotype indicated by white colonies turning to red (**E**) and inability of GuHCl cured cells to grow on *-*Ade media (**F**) where SupC∆NM acts as a control.

A small percentage of GAFQ-Sup35C expressing cells that exhibited nonsense suppressor phenotype were reverted back to the non-supressor state with each subsequent passage on rich medium. This phenomena was more pronounced at later passages (i.e. >32) where each haploid stably exhibiting nonsense suppression phenotype showed high frequency of metastable behavior i.e. appearance of many red colonies in the progeny after streaking (Figure 3A-D). However, this red and white colored colony phenotype was again subsequently maintained stable, generation after generation, upon re-streaking individual colonies picked up from the progeny. Importantly, the white colored colonies from the progeny exhibited nonsense suppression [*PSI*^*+*^] like phenotype (Figure 3E) as they could grow on -Ade medium (Figure 3F), which was reversible by growth in the presence of GuHCl (Figure 3E, F).

**Figure 4:**
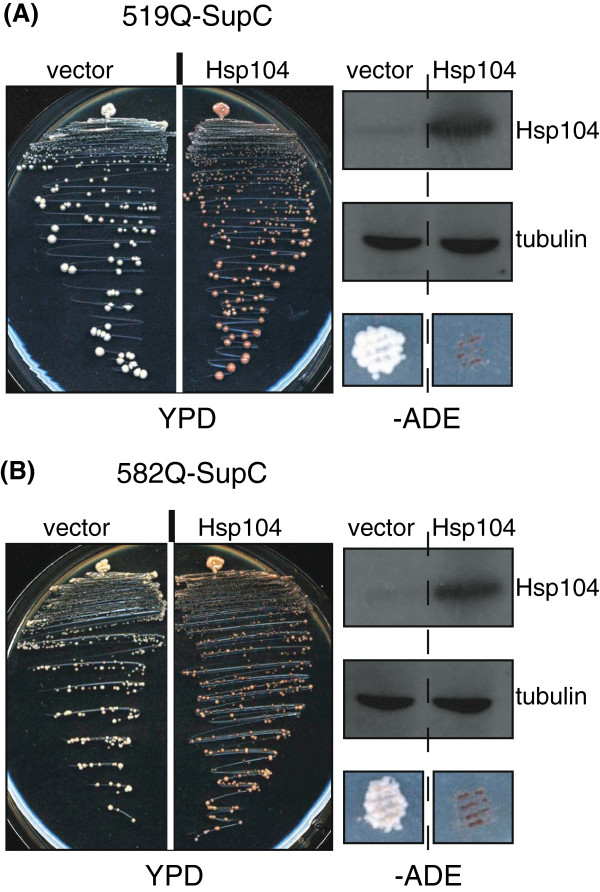
**Over-expression of Hsp104 cures the nonsense suppression prion-like phenotype due to over-expression of GAFq-SupC fusions.** (**A**) 519Q-SupC and (**B**) 582Q-SupC nonsense suppression phenotype is shown to be cured by over-expression of Hsp104, as a control vector alone did not show any effect on prion-like phenotype of GAFQ-SupC cells. In both (**A**) and (**B**), the right panels show western blots of total lysates from cells over-expressing Hsp104 as well as vector alone with anti-Hsp104 (top) and anti-tubulin antibody (middle). Similar to GuHCl experiment, the cells over-expressing Hsp104 lose the ability to grow on *-*Ade plates as compared to vector control (bottom).

The curing of [*PSI*^*+*^] nonsense suppression phenotype may be achieved through inactivation of Hsp104 by GuHCl treatment [37,38] or by Hsp104 mutation or by over-expression of Hsp104 [39]. Indeed the nonsense suppression prion-like phenotype caused by the GAFQ-Sup35C fusions was also cured by over-expression of Hsp104. When compared to control (vector alone) cells, Hsp104 over-expressing cells appeared red on rich medium (YPD) and were unable to grow on -Ade medium, indicative of a curing of 519Q-SupC and 582Q-SupC nonsense suppression phenotype (Figure 4).

**Figure 5:**
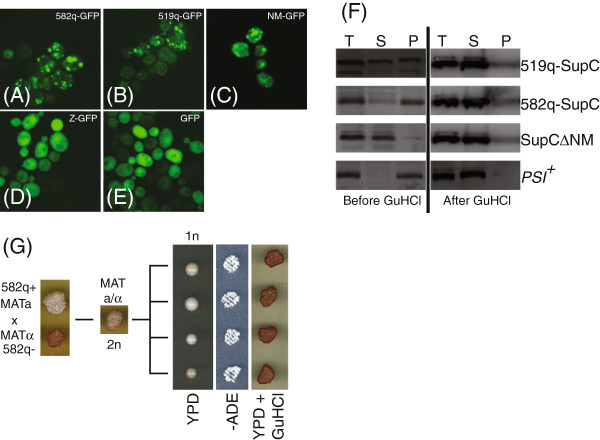
**(A-E) Visualization of protein aggregates from GAF-Q domains fused to GFP.** 582q-GFP (**A**) and 519q-GFP (**B**) clearly show aggregates similar to (**C**) NM-GFP i.e. prion-forming domain of Sup35 (NM region of SUP35) fused to GFP, which was used as a positive control. (**D**) Z-GFP, which contains GFP fused to polyglutamine domain of *Drosophila* Zeste protein, acts as a negative control together with yeast cells containing GFP alone (**E**), which does not show any aggregation. (**F**) Sedimentation analysis of 519Q-SupC, 582Q-SupC expressing cells which exhibit stable non-sense suppression phenotype. Total extracts from GuHCl treated (after GuHCl) or non-treated (before GuHCl) cells expressing 519Q-SupC, 582Q-SupC, SupC∆NM and stable [*PSI*^*+*^] strain were subject to 50 K x g ultacentrifugation for 15 minutes. Total (T), supernatant (S) and pellet (P) fractions were resolved by SDS-PAGE and Western blot was performed using anti-SUP35 serum. Extracts from SupC∆NM and [*PSI+*] strains were used as controls. As with [*PSI+*], both 519Q-SupC, 582Q-SupC exhibit sedimentation in pellet before GuHCl treatement and 519Q-SupC, 582Q-SupC became soluble in supernatant (S) fraction after GuHCl treatement. SupC∆NM lacking prion domain remains soluble regardless of GuHCl treatment. (**G**) Non-mendelian inheritance of *582Q+*. A diploid (2n) made by mating *582q +* MATa strain with 582Q*-* MATα displayed *582Q +* phenotype. Tetrad dissection of diploid (2n) shows 4 meiotic progeny (1n) which stably exhibit non-sense suppression visible by growth on -ADE and all this progeny was curable when grown on YPD containing 5 mM GuHCl.

We determined if we could visually detect different aggregative states of GAF-Q fusion proteins using previously employed fluorescent microscopy techniques for yeast prion characterization (35, 36). We fused the GAF519 and GAF582 Q domains to green fluorescent protein (GFP) under an inducible promoter (Additional file 2: Figure S2A). The prion-forming domain of Sup35 (NM region of Sup35) fused to GFP (NM-GFP), shown to form punctate aggregates in [*PSI*^+^] cells [28,40], was used as a positive control. The GAFQ-GFP and NM-GFP constructs were transformed in four different yeast strains. These vary in strength of the prion phenotype due to presence or absence of either one of [*PSI*^*+*^] and [*PIN*^*+*^] or both prions. GFP is normally soluble in yeast, but as expected the fusion to the NM domain conferred for GFP a capacity to exist in distinct states: a few large aggregates or a soluble protein (Figure 5A-E; Additional file 2: Figure S2B). As described for previously characterized prion determining regions of yeast proteins [22,23,28,29,34,40-43], both the 519Q and 582Q also conferred upon GFP a capacity to exist in distinct states and showed aggregation pattern similar to NM-GFP (Figure 5A-C and Additional file 2: Figure S2B). Like NM-GFP, the aggregation of the GAF-GFP fusions was dependent on the presence of [*PIN*^*+*^], the prion form of the RNQ1 protein of yeast required for Sup35 aggregation and prion formation [28]. Inducible expression of both 519Q-GFP and 582Q-GFP showed aggregation (Figure 5A, B) in 12-15% cells. We also used Q-rich region of *Drosophila* zeste protein (30% Q residues from amino acid 152–432) [44] fused to GFP to monitor if any Q-rich region may lead to aggregation of GFP independent of [*PSI*^*+*^] or [*PIN*^*+*^]. The inducible expression of zeste-GFP (Z-GFP) in any of the yeast strains mentioned above showed no aggregation (Figure 5D and data not shown), which is similar to the pattern exhibited by GFP alone (Figure 5E and Additional file 2: Figure S2B). This reveals that aggregation patterns observed for GAFQ-GFP, similar to NM-GFP (Figure 5A-C and Additional file 2: Figure S2B), is specific, further substantiating that GAF-Q domains exhibit prion-like behavior in yeast. We also employed differential centrifugation assay to explore the possibility of aggregation of 519Q-SupC and 582Q-SupC fusions that exhibited non-sense suppression phenotype in sup35∆ haploids. Total cell extracts from 519Q-SupC and 582Q-SupC expressing cells (white/pink cells), prepared under non-denatured conditions, were fractionated by high speed centrifugation. Supernatant (S) and pellet (P) fractions were probed with anti-Sup35 antibody (Figure 5F). Unlike SupC alone (SupC∆NM), both 519Q-SupC and 582Q-SupC were present in insoluble (pellet) fraction primarily (Figure 5F), which is similar to the behavior of Sup35 protein in a [*PSI*^*+*^] strain (Figure 5F). Moreover, 519Q-SupC was always equally present in both supernatant and pellet fractions. However, GuHCl treated populations of same cells revealed 519Q-SupC and 582Q-SupC in soluble (S) fraction (Figure 5F), which is also similar to the Sup35 protein behavior from [*PSI*^*+*^] cells which were treated with GuHCl (Figure 5F). These results concur with our observations with GAFq-GFP fusions (Figure 5A-E) and corroborate with the notion that 519Q and 582Q domains exhibit prion-like behavior and exist in distinct physical states. Finally, dominant behavior of prion-like GAFQ-SupC (referred as *GAFq+*) was demonstrated by mating *582q +* MATa (pink/white) haploid with a *582q-* MATα haploid, i.e. *582q-* represents a red colony indicating non-prion form of 582q (Figure 5G). The resultant diploid MATa/α (2n) not only displayed *582q +* phenotype (white/pink) but also when sporulated produced haploid (1n) progeny. These exhibited prion like behavior displaying meiotic inheritance of *582q +* phenotype in a dominant, non-Mendelian manner. All 4 haploid (1n) products of meiosis showed stable non-sense suppression phenotype by their growth on -ADE plates, which was curable when grown in the presence of GuHCl (Figure 5G).

By making use of well-characterized genetic assays determining prion-like characteristics of glutamine-rich domains in different proteins, we have identified the Q domains of both the GAF isoforms as prion-like domains. The fusion proteins in which GAF-Q domains were introduced in place of the Sup35p prion domain could support distinct physical and functional prion states that recapitulated the translation termination defect associated with [*PSI*^*+*^]. Importantly, the nonsense suppression prion-like state exhibited by the GAFQ-SupC fusion was cured by growth in the presence of GuHCl. Similar to the [*PSI*^*+*^] prion state of Sup35p, the nonsense suppression phenotype by GAFQ-SupC could also be cured by the over-expression of Hsp104. The GAF-Q domains fused to GFP also formed visible aggregates resembling those of GFP labeled Sup35p in [*PSI*^*+*^], which also depended on [*PIN*^*+*^] [28]. Many sequences with high Q content (as high as that of yeast prions) including human polyglutamine expansion disease proteins, form visible aggregates when over-expressed in yeast as GFP fusions [31,45]. However, only a limited number of Q/N rich sequences are *bone fide* prion domains capable of propagating these aggregates over multiple cell generations even when expressed at low levels [22,23,27,29,34,40]. Construction of a synthetic prion revealed that pathogenically expanded stretch of 62 Qs (Q62) fused to Sup35C or GFP could mimic prion-like behavior, however, 22Q did not show such characteristics [31]. The prion-like behavior of 519Q similar to Sup35 is of significance because it contains only a short Q stretch as compared to Q62. Importantly, computational assessment of GAF519 and GAF582 using prion aggregation prediction algorithm reveals that both proteins have propensity to make prions [46].

As compared to other eukaryotes analyzed, a surprisingly large number of proteins in *Drosophila* have extended Q-rich tracts, remarkably similar to those found in the prion-forming domains of yeast proteins [24-26]. In *in vitro* studies and in transient assays in cell culture fusions of the Q domains with the Gal4 DNA binding domain activate by stabilizing the transcriptional complex [47]. However, in transgenic flies chromatin binding and transcriptional activation activity by GAF was found to be independent of Q domains, leaving open the designation of the exact molecular function [12,17]. So far, the combined results suggest that the Q domains are mostly involved in the formation of larger GAF complexes. The associated prion-like activity might thus provide an ability to GAF to attain distinct conformational states that may be heritable. The high conservation of the C terminal Q-rich domain of GAF in insects, suggests that there is a strong evolutionary preference to maintain such associated structure and function.

## Conclusions

The analysis of GAFQ-SupC fusions in yeast provides an interesting analogy between GAF-Q and the Sup35 prion domain, consistent with the previous findings, revealing that the GAF-Q domain is essential for the formation amyloid fibers *in vitro*[17]. Our results also support the previous findings that oligomerization of GAF found in *Drosophila* cells may be facilitated by the long Q stretches in GAF [19]. We emphasize that GAF may not be a *bone fide* prion but Q domains in GAF may induce conformational switch reminiscent of prion-like behavior. In yeast, prions are not pathogenic but rather act as an epigenetic regulator of cell physiology and several epigenetically heritable traits are found to depend on a prion mechanism [20]. Evidence for regulation of gene expression patterns by propagation of Swi1 and Cy8 proteins as prions has provided a novel link between chromatin remodeling proteins and prion formation [22,23] and it has revealed an additional mechanism for controlling global gene transcription that is based on an inherited self-perpetuating change in the conformation. Our results indicate that the possibility of such an intricate link between chromatin associated proteins, prion formation and epigenetic inheritance of gene expression might also apply in higher eukaryotes. Intriguingly, a large majority of the identified *Drosophila* proteins with Q-rich domains are essential developmental proteins including chromatin regulating proteins from PcG and TrxG involved in epigenetic inheritance [20,26]. It could be envisaged that GAF-Q domains provide an inherent plasticity which may lead to a conformational switch in GAF in a changing environment. Such a Q domain dependent conformation switch in GAF may be regulated by some specific post-translational modifications of GAF and facilitated by molecular chaperones. This could result in modulated gene expression patterns that may contribute to phenotypic variation. We suggest that GAF-Q domain may act as prion-like domain in *Drosophila* and support the notion that oligomeriztaion of GAF and other PcG/TrxG proteins, which is known to be crucial for the function of these proteins, may be facilitated by such prion-like domains [20].

## Methods

### Yeast strains and plasmids used

The genotype and source of different yeast strains used in this study are described in Additional file 3: Table S1. The *sup35*Δ strain, Y133, was generated by transforming strain GT81 [35] with PCR-generated copies of the *kanmx* cassette amplified from plasmid pFA6a-KanMX6 [48] with primers containing regions homologous to the *SUP35* locus (CCATTGTACTGTAACAAAAAGCGGTTTCTTCATGACTTGCTCGGcggatccccgggttaattaa and GCATTTACTTATGTTTGCAAGAAATTTACTCGGCgaattcgagctcgtttaaac, regions homologous to *SUP35* locus indicated in capital letters).

A plasmid containing the functional domain of Sup35, Sup35C, was cloned using primers CCGGCCGCGGATGGTTTGGTGGTAAAGATCACG (forward primer, *Sac II* site underlined) and CCGGGAGCTCTTACTCGGCAATTTTAACAATTTTACC (reverse primer, *Sac I* site underlined) into plasmid p2HG [49], creating plasmid p2H-SupC. The GAF519 and GAF582 regions in the open reading frames (ORF) encoding Q-rich domains were PCR amplified using specific primer pairs which amplified 248 bp and 473 bp products corresponding to positions 1309–1557 in GAF519 ORF and 1270–1743 GAF582 ORF, respectively. Following primer pairs for GAF519-Q (Forward- CGCGGATCCTGATGCAAGGTGTGCT, Reverse- TATTATCCGCGGCTGCGGCTGCGGCTGTT) and GAF582-Q (Forward- AAGAAGGATCCATGGATGCCCAGCAA and Reverse-TATTATCCGCGGGAGAGTCTGTTGTGTTTG) were used where Bam H *I* in the forward and Sac *II* in the reverse primers are underlined. The PCR amplified products were cloned in frame (using Bam H *I* and Sac *II* sites) with C terminal part of Sup35 lacking the N-terminal prion-forming region of Sup35 in plasmid p2H-SupC with *His*^*+*^ selectable marker to generate *p2H-GAFQ-SupC*. The *p2H-GAFQ-SupC* plasmids (and control plasmid p2H-SupC) were transfected in the [*PSI*^*+*^] *sup35*Δ diploid yeast strain Y133 and transformants were selected at *–His* plates. Individual diploids were spotted on medium lacking *His* and replicated plated on plates with YPD and -Ade to monitor prion phenotype. In addition, GAF519-Q and GAF582-Q PCR products with Bam *H* I and Sac II restriction sites, described above, were also cloned in frame with GFP at the C terminus under a copper inducible promoter in the *pCUP-GFP* plasmid [40]. URA was used as selection marker to generate *pCUP-GAFQ-GFP* for visualizing GAFQ-GFP fusions using confocal microscopy. The plasmid pCUP-SUP35NM-GFP [40] expressing the prion domain of Sup35 fused to GFP was used as a positive control.

### Induction of sporulation and isolation of haploids

The *SUP35/sup35Δ* diploid strain Y133, transformed with plasmids p2H-*GAF519Q-SupC*, p2H-*GAF582Q-SupC* or p2H-*SupC* was induced to sporulate and random haploid spores were isolated per standard yeast methods [50]. Single colonies were isolated and transferred onto YPD master plates which were then replica plated on media with G418 and also plates lacking *His* to confirm *sup35Δ* as well as the presence of p2H-*GAF519Q-SupC*, p2H-*GAF582Q-SupC* or p2H-*SupC* plasmids. In addition, same constructs of GAFQ-SupC fusions and SupC alone but with *Ura* as selectable marker were also used to replicate results seen with *His* plasmids. The mating type of individual haploids was determined following standard protocols [50] and haploids with deletions of endogenous *SUP35* was validated by western blotting with antibody which recognize only the N-terminal portion of Sup35 (gift from S.Lindquist). The expression of GAFQ-SupC fusion proteins was confirmed with an antibody which specifically recognizes the C terminus of Sup35 (gift from D. Bedwell). Individual haploids expressing GAF582SupC with *His* (MATa) and *Ura* (MATα) markers were used for mating to generate MATa/α diploid which were selected on –His –Ura plates. Sporulation was induced in these diploids using and tetrad dissection was performed using standard methods [50].

### Western blotting

After overnight growth of cells in 5 ml selective medium, 4 ml culture was transferred to 20 ml fresh medium and incubated at 30°C for 3 hours. Cells were harvested by centrifuging at 4000 rpm for 10 minutes and resuspended in 200 μl of 0.1 M NaOH and incubated at room temperature for 10 minutes. Finally cells were centrifuged at maximum speed for 1 minute, resuspended in SDS gel loading buffer and heated at 95°C for 5–10 min prior to SDS-PAGE analysis.

### Curing of GAFQ-SupC nonsense suppression phenotype

Individual yeast haploids expressing GAFQ-SupC fusions in the *sup35Δ* background that exhibit nonsense suppression phenotype by growth on medium lacking Ade were replica plated on YPD alone and YPD supplemented with 5 mM guanidine HCl. Cells on YPD and YPD plus guanidine HCl were grown for 2 days and kept at 4°C for 2 additional days before comparing their colors. The Ade + cells were also cured by over-expressing Hsp104 by transforming cells with a pGPD-HSP104 plasmid (a high copy plasmid with constituative Hsp104 expression and Ura selectable marker). Transformants were confirmed to have lost Ade + phenotype by growth on medium lacking Ade and color assay was performed by growth on YPD plates compared to vector control cells. Cells cured by over-expressing Hsp104 were analyzed by western blot for expression of Hsp104 and compared to control cells.

### Visualizing GFP-expressing yeast cells

GAFQ-GFP fusions under copper inducible promoters described above were transformed into 74-D694 [51] strains (OT60, OT56, OT55 and GT17) described in Additional file 3: Table S1. The NM-GFP fusion under a copper inducible promoter was used as a positive control to visualize aggregation patterns in these strains [40]. Transformed cells were grown on -Ura plates for 3 days at 30°C. At least 14 individual colonies for each construct were plated onto -Ura master plate which was replica plated onto YPD, YPG, and –Ade plates to monitor color pigmentation, petite phenotype and growth on -Ade, respectively. A single colony of each strain and construct combination was grown overnight in 5 ml -Ura medium as a pre-culture. The overnight culture (100–300 μl) was diluted into fresh 5 ml -Ura medium with 150 μM CuSO_4_ to induce expression of GFP constructs for 12–16 hours. Cells were fixed by directly adding formaldehyde in the culture (final concentration 3.7%) and incubated for additional 20 minutes at room temperature. Cells were harvested by centrifuging at 5000 rpm for 5 minutes 10 ml PBS. The PBS washed cells were spun down and finally resuspended in buffer containing K_2_HPO_4_ (86.6 mM), KH_2_PO_4_ (13.4 mM) and sorbitol (300 mM) and GFP was visualized using a Leica SP2 confocal microscope.

### Centrifugation analysis

Total cell lysates were prepared from GuHCl treated and non-treated log phase yeast cells, expressing GAFQ-SupC fusions, SupC∆NM, and OT55 [*PSI*^*+*^] strain as described [40]. Lysates prepared in non-denaturing condition were fractionated into supernatant and pellet fractions as described [40] and resolved on 10% SDS-PAGE followed by an immunoblot using anti-Sup35 antibody which only recognize C terminus of Sup35.

## Competing interests

The authors declare that they have no competing interests.

## Authors’ contributions

MT and RP conceived the idea. MT, RW, SA performed experiments and MT, RW designed the project. MT, RW, and RP wrote the manuscript. All authors have read and approved the final manuscript.

## Supplementary Material

Additional file 1: Figure S1 The *sup35*∆ deletion in all the haploids analyzed in Figure 2 was confirmed by Western blot analysis of total lysate from each haploid probed with anti-Sup35 antibody, which recognizes only the N-terminal of *SUP35*. Lane 5 shows total lysate from diploid yeast as a positive antibody control. Click here for file

Additional file 2: Figure S2 Visualization of protein aggregates with GFP fused to GAF-Q domains (GAFQ-GFP). (A) Schematic illustration of GAF519 and GAF582 Q domains (GAF-Q) fused to GFP under a promoter inducible with copper (*CUP1*p). The prion-forming domain of Sup35 (NM region of SUP35) fused to GFP was used as a positive control. (B) The constructs shown in (A) were transformed in four different yeast strains which vary in strength of prion phenotype due to presence or absence of either one of [*PSI*^*+*^] and [*PIN*^*+*^] or both prions. Both 519Q-GFP and 582Q-GFP fusions showed aggregation pattern similar to NM-GFP as their aggregation seem to depend on the presence of [*PIN*^*+*^], the prion form of the RNQ1 protein of yeast required for Sup35 aggregation and prion formation. Click here for file

Additional file 3: Table S1 Strains of *S. cerevisiae* used in this study. Click here for file
