# Supplementary figures and images for: Drosophila GAGA factor polyglutamine domains exhibit prion-like behavior
Source: BMC Genomics. 2013 Jun 3;14:374. doi: 10.1186/1471-2164-14-374 (PMC3701498; doi:10.1186/1471-2164-14-374)

Supplementary Figure 1.

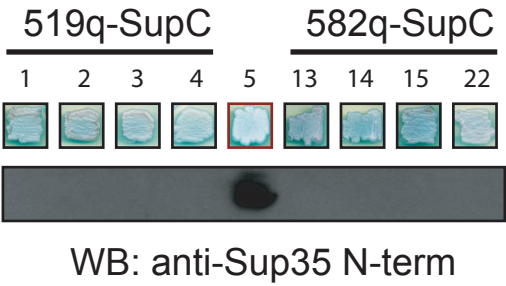

Supplement: Additional file 1: Figure S1 — The sup35∆ deletion in all the haploids analyzed in Figure 2 was confirmed by Western blot analysis of total lysate from each haploid probed with anti-Sup35 antibody, which recognizes only the N-terminal of SUP35. Lane 5 shows total lysate from diploid yeast as a positive antibody control. [file 1471-2164-14-374-S1.pdf]

Supplementary Figure 2

(A)

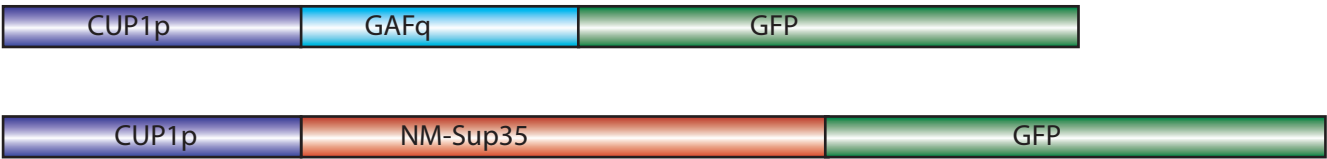

(B)

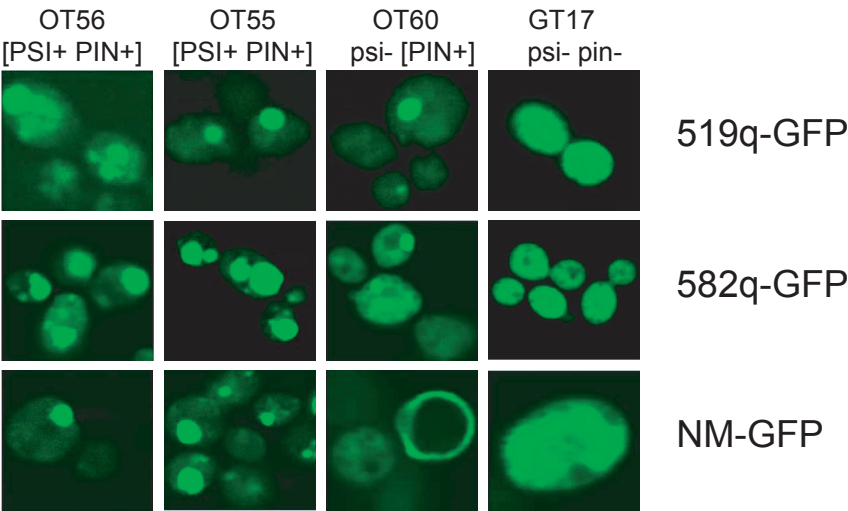

Supplement: Additional file 2: Figure S2 — Visualization of protein aggregates with GFP fused to GAF-Q domains (GAFQ-GFP). (A) Schematic illustration of GAF519 and GAF582 Q domains (GAF-Q) fused to GFP under a promoter inducible with copper (CUP1p). The prion-forming domain of Sup35 (NM region of SUP35) fused to GFP was used as a positive control. (B) The constructs shown in (A) were transformed in four different yeast strains which vary in strength of prion phenotype due to presence or absence of either one of [PSI+] and [PIN+] or both prions. Both 519Q-GFP and 582Q-GFP fusions showed aggregation pattern similar to NM-GFP as their aggregation seem to depend on the presence of [PIN+], the prion form of the RNQ1 protein of yeast required for Sup35 aggregation and prion formation. [file 1471-2164-14-374-S2.pdf]
