# Supplementary material for: Drosophila GAGA factor polyglutamine domains exhibit prion-like behavior
Source: BMC Genomics. 2013 Jun 3;14:374. doi: 10.1186/1471-2164-14-374 (PMC3701498; doi:10.1186/1471-2164-14-374)
Supplement: Additional file 3: Table S1 — Strains of S. cerevisiae used in this study. [file 1471-2164-14-374-S3.pdf]

Supplementary Table 1. Strains of *S. cerevisiae* used in this study.

Strain	Genotype	reference
**OT60 ([psi]-74-D694)**	MATa ade1-14 his3 leu2 trp1 ura3 [psi PIN+]	(1)
**OT55 ([PSI+]1-1-74-D694)**	MATa ade1-14 his3 leu2 trp1 ura3 [PSI+ PIN+] weak [PSI+] derivative of OT60	(1)
**OT56 ([PSI+]7-1-74-D694)**	MATa ade1-14 his3 leu2 trp1 ura3 [PSI+ PIN+] strong [PSI+] derivative of OT60	(1)
**GT17 ([psi- pin-])**	MATa ade1-14 his3 leu2 trp1 ura3 [psi pin]	(2)
**GT81**	MATa/α ade1-14 his3-Δ200(or 11, 15) leu2.3, 112 trp1-Δ ura3-52 lys2	(3)
**Y133**	MATa/α ade1-14 his3-Δ200(or 11, 15) leu2.3, 112 trp1-Δ ura3-52 lys2 sup35::kanmx; Δsup35	This study

1. Derkatch IL, Chernoff YO, Kushnirov VV, Inge-Vechtomov SG, Liebman SW (1996) Genesis and variability of [PSI] prion factors in Saccharomyces cerevisiae. *Genetics* 144: 1375-1386.
2. Derkatch IL, Bradley ME, Zhou P, Chernoff YO, Liebman SW (1997) Genetic and environmental factors affecting the de novo appearance of the [PSI+] prion in Saccharomyces cerevisiae. *Genetics* 147: 507-519.
3. Chernoff YO*, et al.* (2000) Evolutionary conservation of prion-forming abilities of the yeast Sup35 protein. *Mol Microbiol* 35: 865-876.
